# Supplementary material for: Animal Cellulose with Hierarchical Structure Isolated from Halocynthia aurantium Tunic as the Basis for High-Performance Pressure-Resistant Nanofiltration Membrane
Source: Membranes (Basel). 2022 Oct 6;12(10):975. doi: 10.3390/membranes12100975 (PMC9607593; doi:10.3390/membranes12100975)
Supplement: Supplementary file 1 [file membranes-12-00975-s001.zip › membranes-1945830-supplementary.pdf]

Supplementary material

Animal cellulose with hierarchical structure isolated from *Halocynthia aurantium* tunic as the basis for high performance pressure resistant nanofiltration membrane

Svetlana V. Kononova*, Albert K. Khripunov*, Vladislav N. Romanov**, Anton S. Orekhov***, Alexey A. Mikhutkin****, Elena N. Vlasova*, Maxim S. Lukasov***, Vera V. Klechkovskaya***

*Institute of Macromolecular Compounds, Russian Academy of Science, Bolshoy pr. 31, 199004 Saint-Petersburg, Russia

** Zoological Institute, Russian Academy of Sciences, Universitetskaya emb., 1, St.-Petersburg, 199034, Russia

***FSRC "Crystallography and Photonics" RAS, Leninsky Prospekt 59, Moscow 119333, Russia

****National Research Centre "Kurchatov Institute", pl. Kurchatova, 1, Moscow, 123098, Russia

1. Digital images of the upper and lower surface layers of Cellokon-AKH

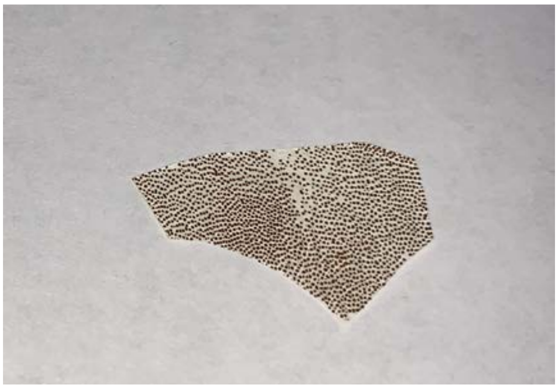

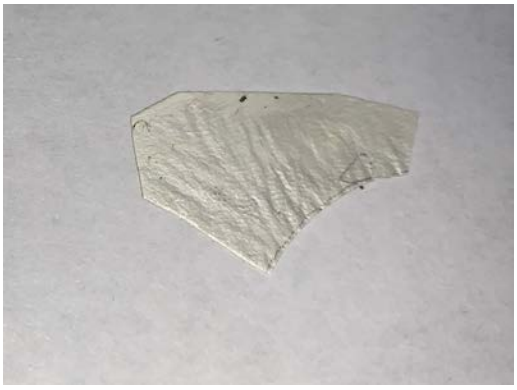

2. Photogrammetry

The binocular vision of humans and animals is based on this effect: a spatial representation of an object is obtained on the basis of two images (from the right and left eyes) obtained from different viewpoints (at different angles). Based on these differences, the brain forms a spatial (volumetric) perception of the observed object (stereo effect). The images obtained in this way are called stereo images (or in the case of two images, a stereo pair). The classic method for creating stereoscopic images is photogrammetry. This method is widely used in aerospace photography for mapping and measuring the topography of the Earth's surface.

3. Profiles. Model building

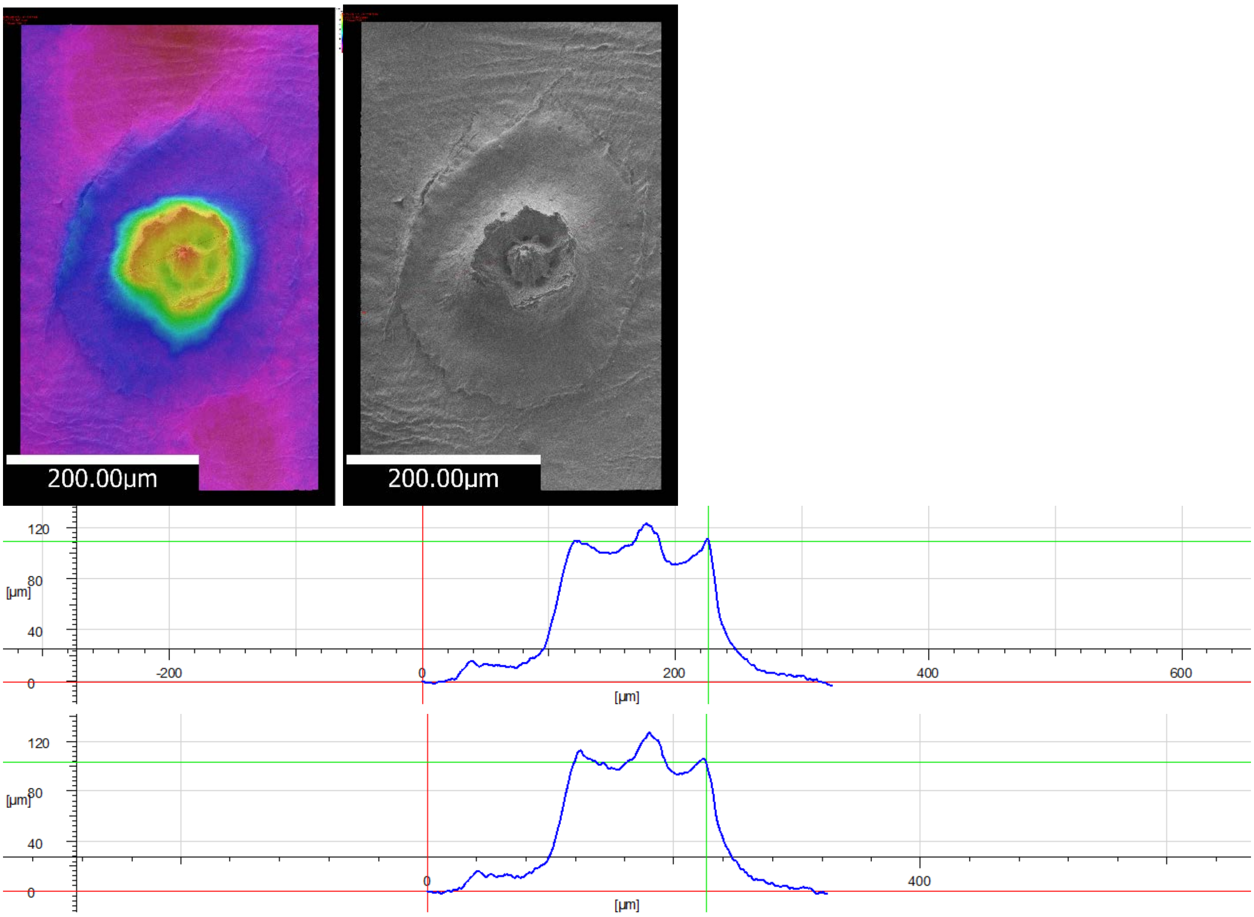

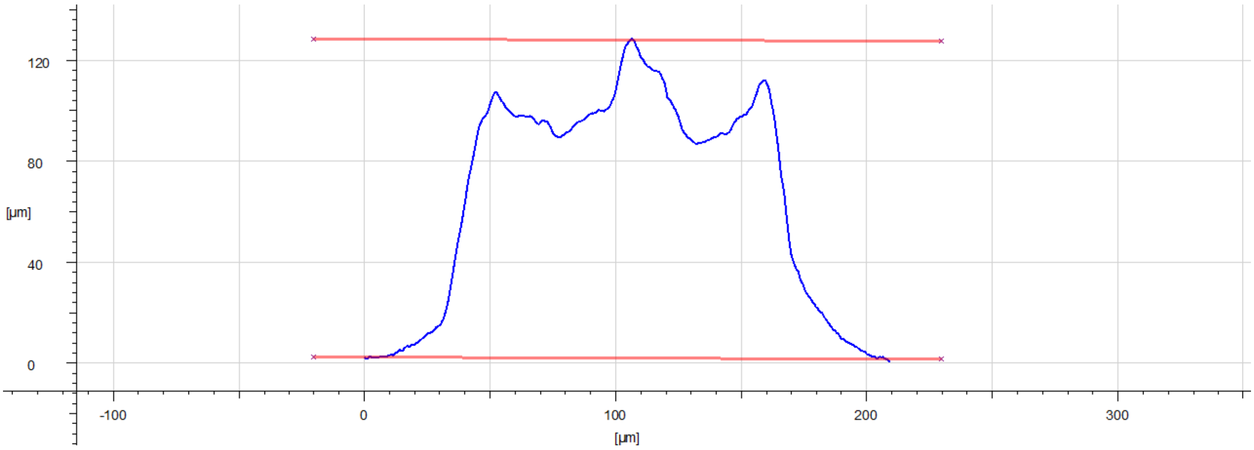

Высота до 127 мкм

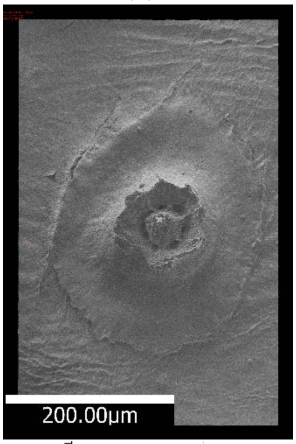

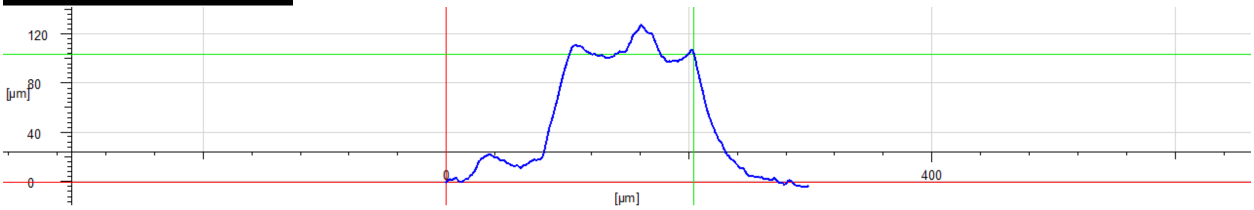

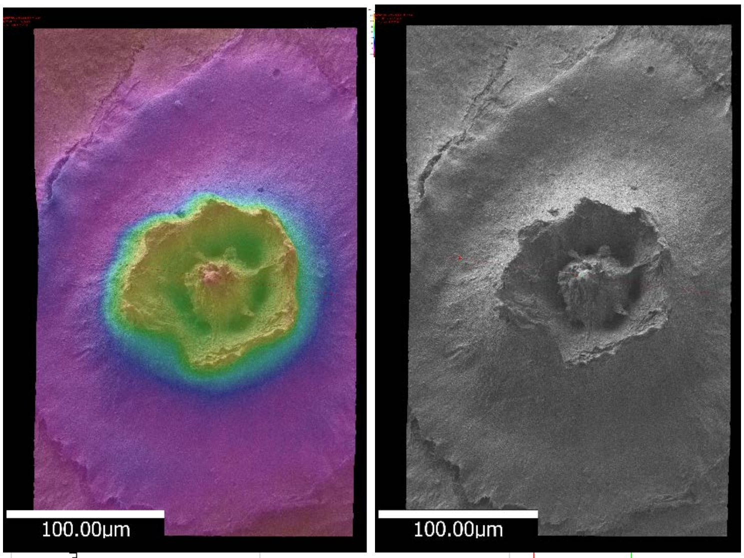

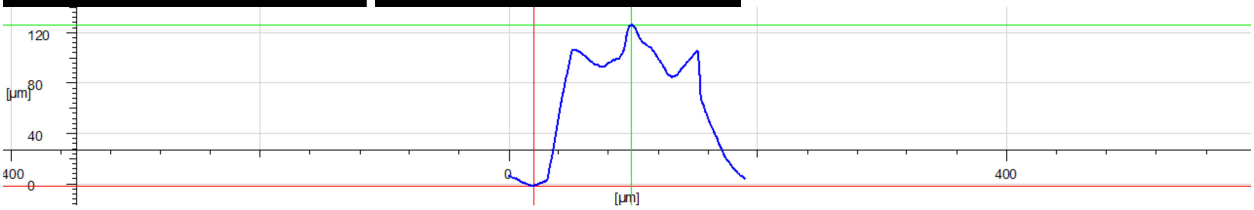

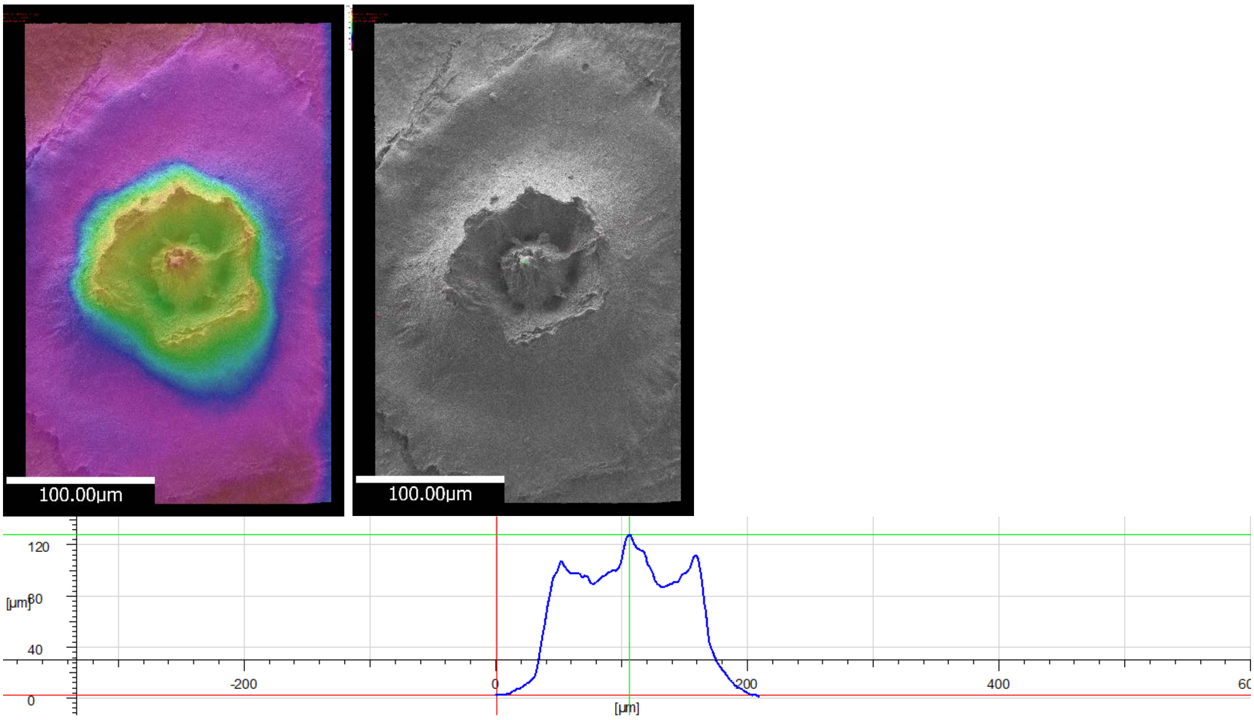
100.00µm
100.00µm
120
80
40
0
[µm]
-200
0
200
400
[µm]

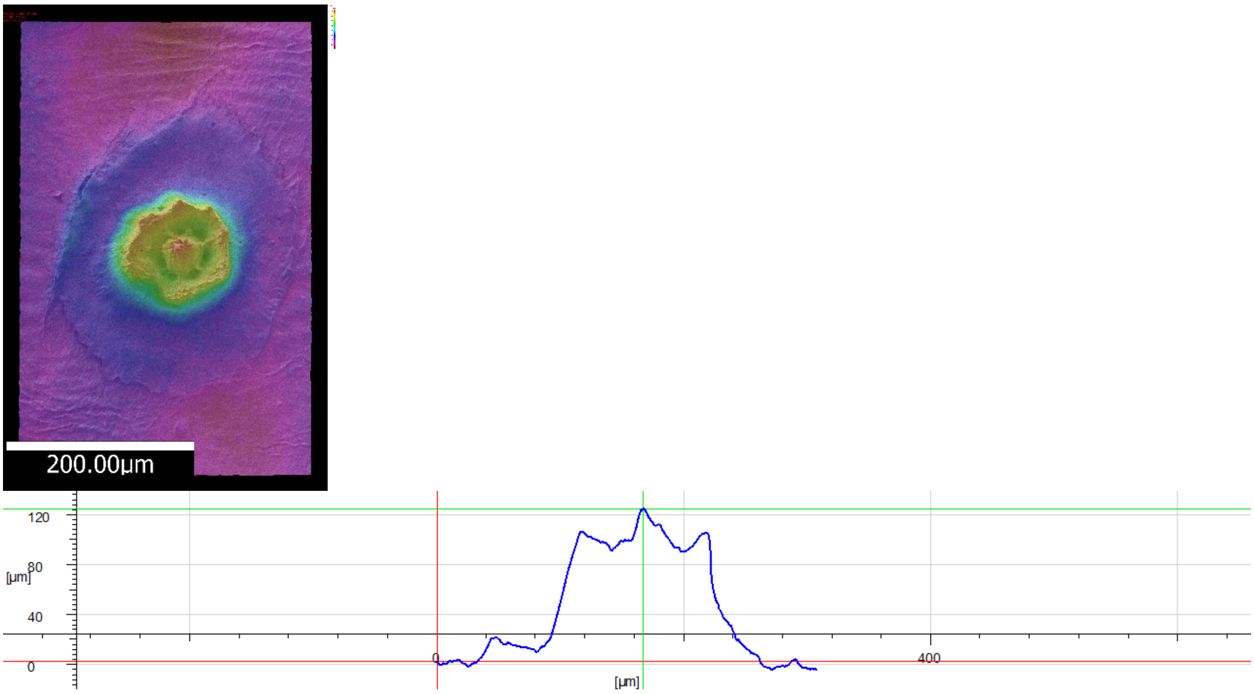
200.00µm
120
80
40
0
[µm]
0
400
[µm]

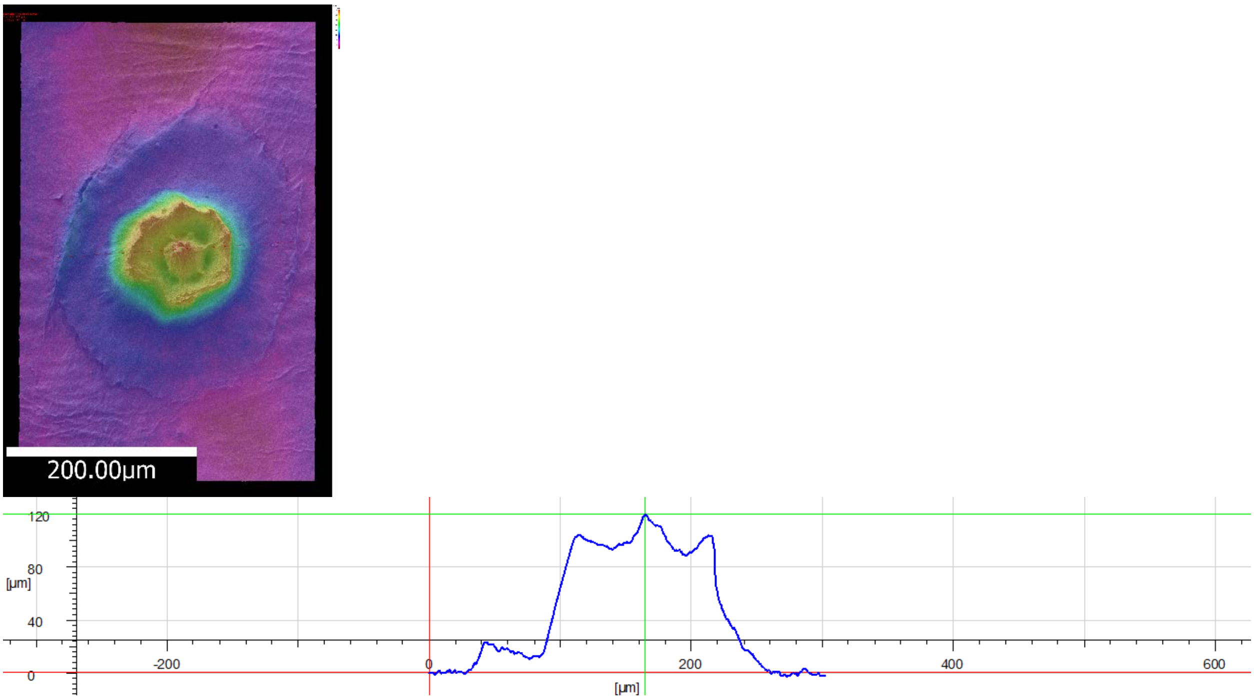
200.00µm
120
80
[µm]
40
0
-200
0
200
400
600
[µm]

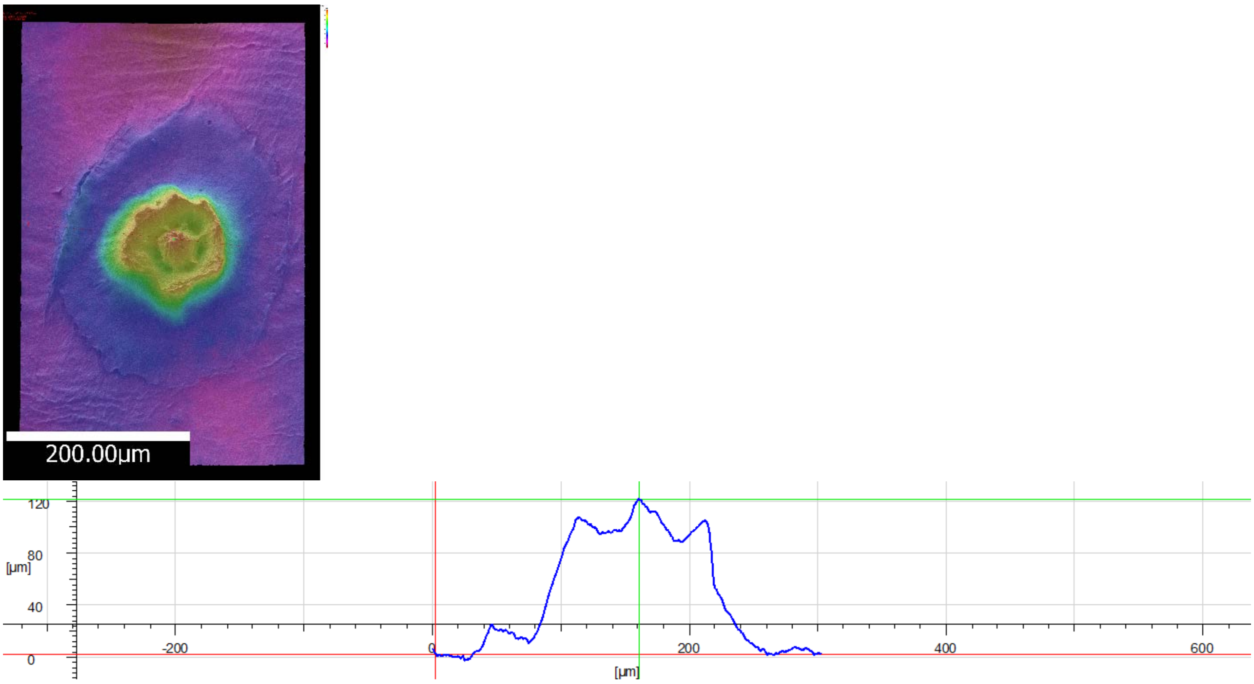
200.00µm
120
80
[µm]
40
0
-200
0
200
400
600
[µm]

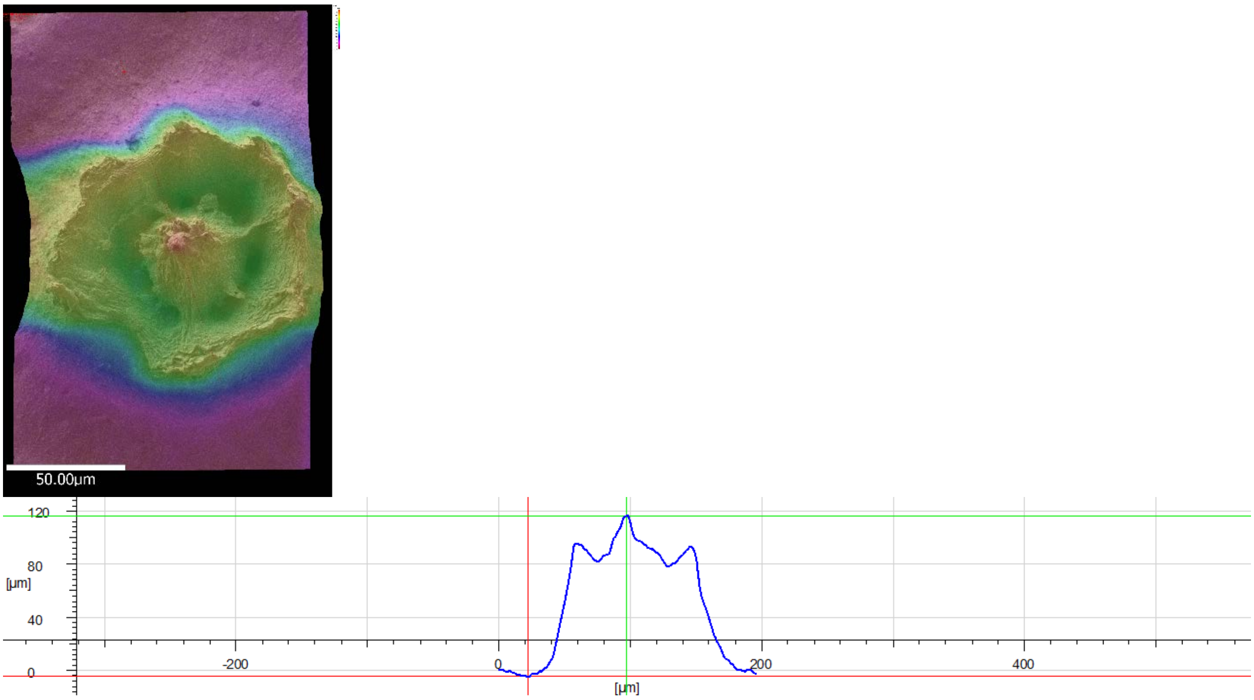
50.00µm
120
80
[µm]
40
0
-200
0
200
400
[µm]

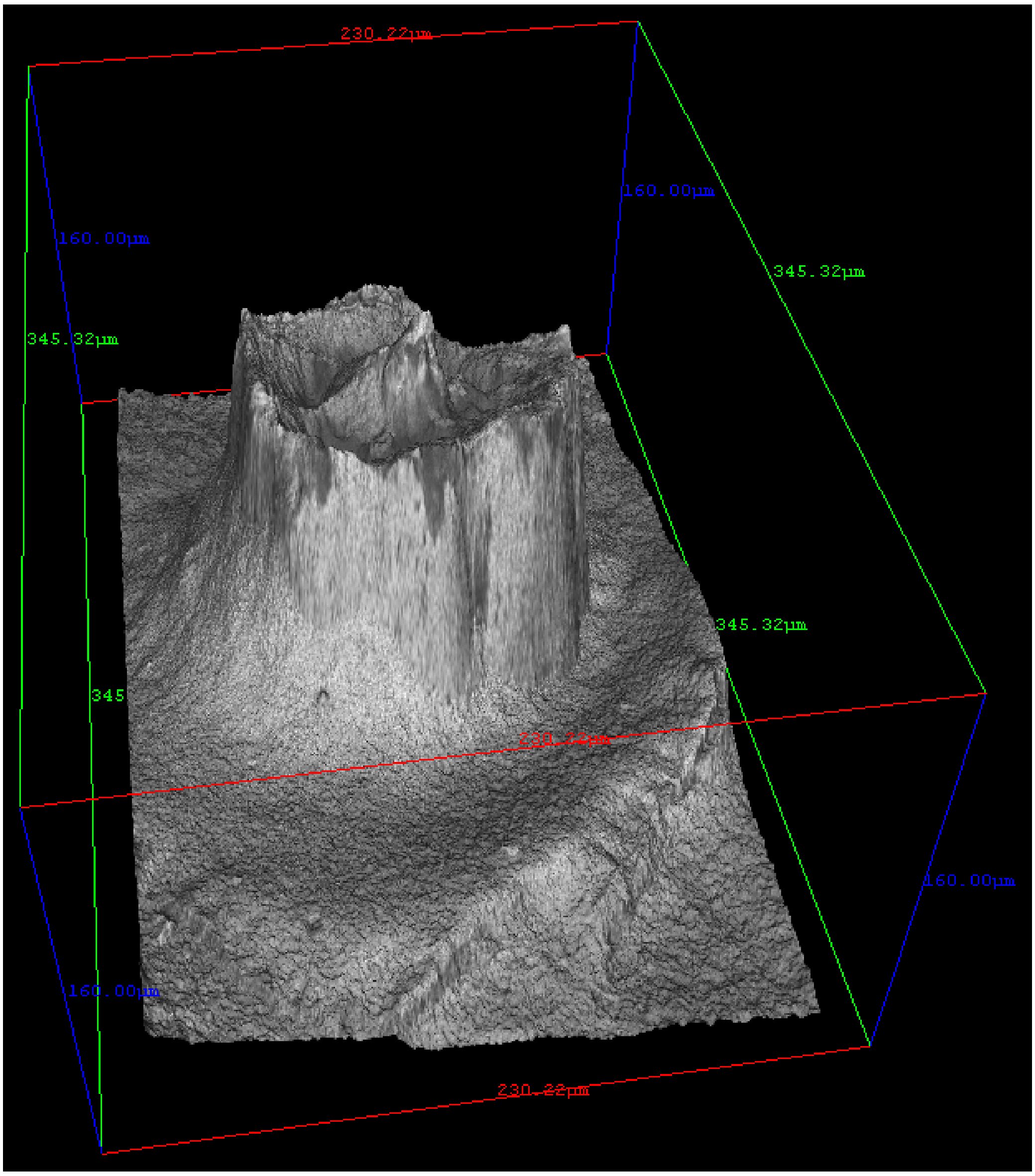
230.22µm
160.00µm
345.32µm
160.00µm
345.32µm
345.32µm
345
230.22µm
160.00µm
160.00µm
230.22µm
